# Evaluation of systemic and cerebral hemodynamics after systematic and early extracorporeal cardiopulmonary resuscitation in swine^☆^

**DOI:** 10.1016/j.resplu.2026.101233

**Published:** 2026-01-18

**Authors:** Julian San Geroteo, Ali Jendoubi, Fanny Lidouren, Naoto Watanabe, Yara Abi Zeid Daou, Alice Hutin, Lionel Lamhaut, Nadir Mouri, Bijan Ghaleh, Pierre-Louis Léger, Jerome Rambaud, Rebecca Goutchtat, Matthias Kohlhauer, Renaud Tissier

**Affiliations:** a Univ Paris Est Créteil, INSERM, IMRB, F-94010 Créteil, France; b Ecole Nationale Vétérinaire d’Alfort, IMRB, AfterROSC Network, F-94700 Maisons-Alfort, France; c Pediatric Intensive Care Unit, Robert-Debré Mother-Child University Hospital, Paris, France; d SAMU de Paris, Necker University Hospital, Assistance Publique-Hôpitaux de Paris, Paris, France; e APHP, Hôpitaux Universitaires Henri Mondor, Département de Biochimie-Pharmacologie, 94000 Creteil, France; f Neonatal and Pediatric Intensive Care Unit, Armand-Trousseau Children’s Hospital, Paris, France

**Keywords:** Cardiac arrest, Extracorporeal cardiopulmonary resuscitation, Epinephrine, Cerebral hemodynamics, Brain injury

## Abstract

**Background:**

Extracorporeal cardiopulmonary resuscitation (ECPR) is thought to be efficient when performed promptly after cardiac arrest. However, its neurological benefit remains questionable if applied very early and systematically. Accordingly, we sought to compare systemic and cerebral hemodynamics when ECPR was implemented systematically compared to conventional cardiopulmonary resuscitation (CCPR) with epinephrine.

**Material and methods:**

Following 5 min of untreated ventricular fibrillation, pigs were randomly submitted to CCPR with epinephrine or crystalloid-primed ECPR after either a 10- or 30-min low-flow (4 groups: CCPR 10′, ECPR 10′, CCPR 30′ and ECPR 30′. Defibrillations were then delivered until the return of spontaneous circulation (ROSC). Swine were followed 240 min from cardiopulmonary onset.

**Results:**

Six pigs were included in each group. Survival rate was higher in CCPR 10′ group vs ECPR 10′ (6/6 vs 2/6; *p* = 0.02) but not significantly different between CCPR 30′ and ECPR 30′ groups (2/6 vs 0/6; *p* = 0.53). In ECPR 10′ and 30′ groups, ECPR was associated with lower cerebral perfusion pressure, lower jugular venous oxygen saturation and higher-pressure reactivity index after ROSC, as compared to CCPR 10′ and 30′. A decrease in mean arterial pressure, along with an increase in norepinephrine dose and blood lactate level were also found in ECPR 10′ and 30′ groups after ROSC, as compared to CCPR 10′ and 30′.

**Conclusions:**

The early and systemic implementation of ECPR after either a 10- or 30-min low-flow was associated with impaired cerebral and systemic hemodynamics after ROSC, as compared to CCPR with epinephrine.

## Introduction

Cardiac arrest (CA) carries a grim prognosis, with a 1-month survival rate of only 10.7% among patients receiving cardiopulmonary resuscitation (CPR) in out-of-hospital setting.^1^ Following return of spontaneous circulation (ROSC), the onset of hypoxic brain injury is a cornerstone,^2^ representing the leading cause of mortality^3^ and long-term sequelae among survivors.^4^ Consequently, a primary objective of CPR is the preservation of cerebral function and the mitigation of brain injury.^4^ After starting basic life support (BLS), which includes high-quality chest compressions and prompt defibrillation for shockable CA, two distinct advanced life support (ALS) approaches can be employed. The first is conventional cardiopulmonary resuscitation (CCPR), which could involve, among others, the administration of epinephrine every 3–5 min for non-shockable CA in conjunction with continued BLS measures.^5^, ^6^ The second is extracorporeal cardiopulmonary resuscitation (ECPR), defined as the rapid-deployment of veno-arterial extracorporeal membrane oxygenation (ECMO) during CA to provide circulatory and respiratory support.^7^ Current international guidelines advocate considering ECPR as a rescue therapy for selected patients when CCPR is failing, if such procedure may be established less than 60 min after CPR start.^5^, ^6^ This sequential approach raises several concerns. On the one hand, epinephrine is sometimes considered as ineffective for long-term outcomes or even deleterious for acute brain injury,^3^, ^8^ which could explain its association with reduced mortality but no significant improvement in favorable neurological survival as compared with placebo.^9^, ^10^ On the other hand, while the use of ECPR is rapidly expanding in cases of prolonged CA, its effect on long-term survival with favorable neurological outcomes is still debated.^11^, ^12^, ^13^ Thus, although the optimal time to initiate such a procedure is still uncertain,^5^, ^6^ it is often hypothesized that the benefits of ECPR could be enhanced if applied systematically and earlier in CPR management, as opposed to CCPR. However, the neurological impact of a systematic early ECPR strategy (specifically within the first 30 min of CA) is unknown to date, since current experimental models of ECPR hardly reflect clinical practice with prolonged no-flow time or lack of low-flow time before ECPR start.^14^

Accordingly, the present study aimed at evaluating the effects of early and systematic ECPR after CA. We assessed its effect on both systemic and cerebral hemodynamics and oxygenation compared to CCPR. To achieve this goal, we used a swine model of CA with a 5-min no-flow followed by a 10- or 30-min low-flow before advanced care with ECPR or CCPR.

## Methods

The study protocol was reviewed and approved by the ethical committee ComEth Anses-EnvA-UPEC (Committee No. 16, project #47825-2024022914398265 v4). All procedures were conducted in accordance with the ARRIVE guidelines^15^ and the European Community Standards on the Care and Use of Laboratory Animals.

### Animal preparation

Female Large White/Landrace swine (29–39 kg) were fasted overnight with free access to water. After premedication by zolazepam-tiletamine (10 mg/kg, i.m.) and methadone (0.75 mg/kg, i.m.), pigs were anesthetized by propofol (2 mg/kg bolus, followed by continuous infusion of 10 mg/kg/h, i.v.) and intubated. Mechanical ventilator was set to the following parameters: tidal volume (TV) of 8 ml/kg; positive end-expiratory pressure (PEEP) of 5 cmH_2_O; fraction of inspired oxygen (FiO_2_) of 30%. The respiratory rate (RR) was adjusted to maintain normocapnia, defined as an arterial carbon dioxide partial pressure (PaCO_2_) between 35 and 45 mmHg.

Four vascular catheters were then introduced according to the Seldinger technique. Two catheters were introduced through the right femoral artery (7 Fr) and vein (8 Fr), enabling the insertion of the pacemaker probe and a pressure gauge (*Millar, SPR-524, Houston, TX, USA)* for continuous blood pressure monitoring. The two other catheters were introduced in the right external jugular vein, i.e., one retrogradely (6 Fr) for jugular venous oxygen saturation (SjvO_2_) measurement and the other anterogradely (3-lumen, 7 Fr/16 cm) for continuous right atrial pressure (RAP) recording and drug administration, respectively. After median cervicectomy, a 2.5 mm blood flow probe (*PS-Series Probes, Transonic, NY, USA)* was placed around the left internal carotid artery to monitor carotid blood flow (CBF). A pressure gauge (*Millar, SPR-524, Houston, TX, USA*) was also implanted into the cerebral cortex to monitor intracranial pressure (ICP). In pigs that were allocated to undergo ECPR, two HLS cannulas (*Maquet Cardiopulmonary AG, Rastatt, Germany*) were placed into the left femoral artery (15 Fr) and vein (21 Fr). The ECMO circuit, primed with Ringer’s lactate solution and de-aired before use, consisted of a Deltastream DP3 pump head and console (*Medos Medizintechnik AG, Stolberg, Germany*), a PLS-i Oxygenator and a 3/8 tubing set (*Maquet Cardiopulmonary AG, Rastatt, Germany*). An air-oxygen blender (*Sechrist, Anaheim, Calif*) was connected to the circuit to set sweep gas flow (SGF) and fraction of membrane oxygen (FmO_2_). An infusion of Ringer Lactate (15 ml/kg, i.v.) was administered throughout this phase to compensate for fluid loss. After completing the instrumentation, the animals received an initial administration of unfractionated heparin (100 IU/kg, i.v.). After a period of stabilization, ventricular fibrillation (VF) was induced by a pacemaker probe introduced in the right ventricle through the femoral venous catheter (A/C 10 V) and then left untreated for 5 min. During this no-flow period, anesthesia, crystalloid infusion and mechanical ventilation were discontinued.

### Experimental protocol

**Fig. 1:**
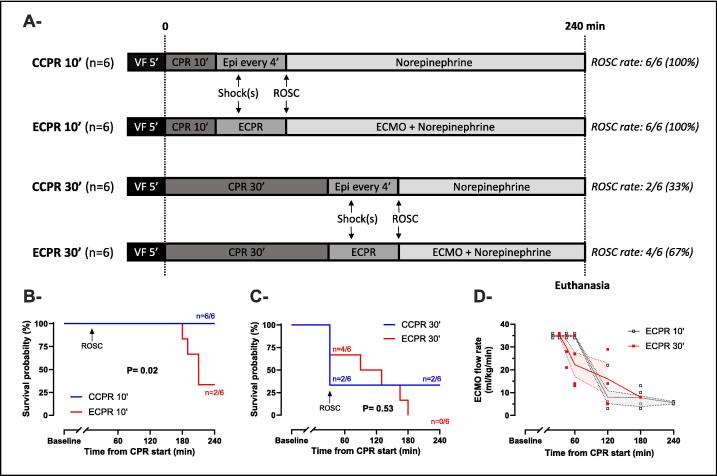
**Experimental protocol (A), probability of survival (B and C) and ECMO flow rate (D)**. Panels B and C: Kaplan-Meier plots of probability of survival between CCPR 10′ and ECPR 10′ groups (B) and between CCPR 30′ and ECPR 30′ groups (C). Analyses were performed with the log-rank test. *P*-value <0.05 was considered statistically significant. Panel D: Symbols represent sample size and dispersion at each time of interest (*t* = 35, 60,120, 180, 240 min from CPR start). Lines and error bands represent means and SEM respectively. No statistical comparisons were performed between groups. *CCPR, conventional cardiopulmonary resuscitation; CPR, cardiopulmonary resuscitation; ECMO, extracorporeal membrane oxygenation; ECPR, extracorporeal cardiopulmonary resuscitation; Epi, Epinephrine; ROSC, return of spontaneous circulation; VF, ventricular fibrillation*.

Fig. 1a summarizes the experimental protocol. After 5 min of no-flow, CPR was initiated with automated chest compressions at a rate of 100/min (*LUCAS 3 device, Stryker Medical, Kalamazoo, USA*) and resumption of mechanical ventilation (TV = 6 ml/kg; RR = 10 breaths/min; PEEP = 5 cmH_2_O; FiO_2_ = 100%). After this period of basic CPR, animals were randomly divided into four experimental groups according to the ALS strategy and to the low-flow period duration when it was initiated: Group 1, CCPR after 10 min low-flow (CCPR 10′); Group 2, ECPR after 10 min low-flow (ECPR 10′); Group 3, CCPR after 30 min low-flow (CCPR 30′); Group 4, ECPR after 30 min low-flow (ECPR 30′). ALS was then provided with modalities varying between groups, i.e., CCPR or ECPR. In CCPR 10′ and CCPR 30′ groups, epinephrine (20 μg/kg, i.v.) was administered every 4 min if coronary perfusion pressure (calculated as the difference between blood pressure and RAP at end-decompression) was below 30 mmHg. In ECPR 10′ and ECPR 30′ groups, arterial and venous cannulas were connected to the ECMO circuit, then ECPR was started with a pump flow of 35 ml/kg/min, without any epinephrine administration. The right position of the cannulas was evaluated by transthoracic echography. It was also confirmed at necropsy. In all 4 groups, defibrillation attempts (7 J/kg) were applied 2 min after the start of ALS and repeated every 2 min until ROSC, which was defined as the occurrence of an organized cardiac rhythm and a mean arterial pressure (MAP) above 40 mmHg for more than 5 min. CPR was stopped if ROSC wasn't achieved after 15 min of ALS. Animals with no ROSC were then considered dead in the survival analysis.

Following ROSC, a mechanical ventilation was applied according to a lung-protective strategy (TV = 6 ml/kg; RR = 20 breaths/min; PEEP = 5 cmH_2_O; FiO_2_ = 30%) in CCPR 10′ and CCPR 30′ groups or a ultra-lung-protective strategy (TV = 4 ml/kg; RR = 10 breaths/min; PEEP = 5 cmH_2_O; FiO_2_ = 30%; SGF = 1 l/min; FmO_2_ = 40%) in ECPR 10′ and ECPR 30′ groups, respectively. Normocapnia was then maintained by adjusting the RR (CCPR 10′ and CCPR 30′ groups) or the SGF (ECPR 10′ and ECPR 30′ groups). A temperature of 37.0 ± 0.5 °C was targeted using thermal pads and infra-red light in all groups. All pigs also received hourly injections of unfractionated heparin (100 IU/kg, i.v.) and methadone (0.75 mg/kg, i.m.).

In the CCPR 10′ and CCPR 30′ groups, norepinephrine infusion was continuously adjusted after ROSC to target a MAP at 70 ± 5 mmHg, with a maximum level set at 5.0 μg/kg/min (i.v.). Ringer Lactate (20 ml/kg, i.v.) was also administered. In case of hypotension despite maximal dose of norepinephrine, fluid administration could be repeated with a maximal tolerable amount of 40 ml/kg for the entire experiment. If hypotension persisted despite norepinephrine and maximal fluid amounts, no additional treatment was attempted, leading to potential premature animal death.

In the ECPR 10′ and ECPR 30′ groups, ECMO flow target was set at 35 ml/kg/min with MAP at 70 ± 5 mmHg. Ringer Lactate was again administered at initial amount of 20 ml/kg, i.v., with the possibility to increase this amount to 40 ml/kg if ECMO flow dropped with MAP decrease. Norepinephrine was also added to achieve the MAP level, with a maximum dose of 5.0 μg/kg/min (i.v.). In case of concomitant hypotension and ECMO flow drop despite maximal fluid and norepinephrine administration, no additional treatment was attempted, potentially leading to animal death before the end of the follow-up. Conversely, if the hemodynamic status was good with MAP spontaneously >80 mmHg, a stepwise reduction of ECMO flow by 10 ml/kg/min was attempted every 15 min until a final flow rate of 5 ml/kg/min could be done. If MAP decreased, ECMO flow target was increased once again, while remaining below 35 ml/kg/min.

After a maximal period of 4 h of follow-up after cardiac arrest, all surviving animals were euthanized (pentobarbital 60 ml/kg i.v.).

### Investigated parameters

Heart rate, invasive arterial blood pressure, RAP, coronary perfusion pressure (CoPP), ICP and CBF were recorded continuously using dedicated software (*Hem, Notocord, France*). Cerebral perfusion pressure (CePP) was defined as the difference between MAP and ICP. To assess cerebral autoregulation, we calculated the pressure reactivity index (PRx), as the correlation coefficient between 40 consecutive 6-s of MAP and ICP, at baseline and after CPR start (*t* = 35, 60, 120, 180 and 240 min). A negative or zero PRx value reflects normally reactive vascular bed whereas a PRx value greater than 0.25–0.30 reflects impaired cerebral autoregulation.^16^ Cardiac index was measured by transthoracic echocardiography at baseline and every hour after CPR start. Arterial, venous and jugular bulb blood gases were collected at baseline and after CPR start (*t* = 35, 60, 120, 180 and 240 min). Blood levels of alanine aminotransferase (ALT), creatinine, troponin I, and protein S-100β (PS-100β) were measured at baseline and after CPR start (120 and 240 min).

### Statistical analysis

Data were expressed as mean ± standard error of the mean (SEM). The normality of the distribution of continuous parameters was verified by the Shapiro–Wilk test. At baseline, continuous variables were compared using Student’s T-test between the CCPR 10′ and ECPR 10′ groups, as well as between the CCPR 30′ and ECPR 30′ groups. A similar test was conducted at *t* = 8 min in the ECPR 10′ vs CCPR 10′ groups and *t* = 20 min In the ECPR 30′ and CCPR 30′ groups, respectively. These timings correspond to the effect of ECPR vs CCPR before putative ROSC. Following ROSC, comparisons between the CCPR 10′ and ECPR 10′ groups were conducted using a mixed-effects model for repeated measures, which assessed time effects, group effects, and time × group interactions. Specific comparisons at individual time points were not performed. For the CCPR 30′ versus ECPR 30′ analysis, the same mixed-effects model was used, except for evaluating time × group interactions due to the limited number of resuscitated animals. Exact *P*-values of the mixed models are reported in Supplementary Material 1: Table S1 (CCPR 10′ vs ECPR 10′) and Supplementary Material 2: Table S2 (CCPR 30′ vs ECPR 30′). After CA, the probability of survival was also analyzed with Kaplan–Meier method and compared between groups with the log-rank test. *P-value* of less than 0.05 was considered statistically significant. Analyses were performed with GraphPad Prism Software (*GraphPad Software, California, USA*).

## Results

### Survival and CPR characteristics

Twenty-four animals were successfully included in the different groups (*n* = 6/group). Baseline and CPR characteristics were similar in the CCPR 10′ and ECPR 10′ groups, as well as in CCPR 30′ and ECPR 30′ groups (Table 1). ROSC was achieved in all pigs in CCPR 10′ and ECPR 10′ groups but only 2/6 (33%) and 4/6 (67%) in CCPR 30′ and ECPR 30′ groups, respectively. In resuscitated animals, time to ROSC was longer in ECPR 30′ vs CCPR 30′ group, along with higher number of defibrillation attempts. on Survival rate at the end of the experiment was: 6/6 (100%) vs 2/6 (33%) in CCPR 10′ and ECPR 10′ groups, respectively, and 2/6 (33%) vs 0/6 in CCPR 30′ and ECPR 30′ groups (Fig. 1b, 1c). As illustrated by Fig. 1d, ECMO flow rate throughout the experiment was below the initial target level of 35 ml/kg/min in both ECPR 10′ (27 ± 13 ml/kg) and ECPR 30′ groups (25 ± 11 ml/kg). This was either related to (1) the early weaning from ECMO in 2/6 pigs (33%) of the ECPR 10′ group or to (2) the impossibility to achieve the ECMO targets in other animals. The latter impossibility was the consequence of fluid administration restriction, which could not exceed 40 ml/kg according to the study design. This also explains post-ROSC deaths due to persistent hypotension in the ECPR groups.

**Table 1:** Baseline and CPR characteristics.

	**CCPR 10′** **(*N* = 6)**	**ECPR 10′** **(*N* = 6)**	**CCPR 30′** **(*N* = 6)**	**ECPR 30′** **(*N* = 6)**
**Baseline characteristics**				
Weight, kg	33.7 ± 1.2	33.7 ± 1.1	32.8 ± 0.8	35.9 ± 0.9
Bladder temperature, °C	37.5 ± 0.3	37.6 ± 0.2	37.6 ± 0.2	38.2 ± 0.3
Carotid blood flow, ml/kg/min	3.9 ± 0.5	3.6 ± 0.5	3.7 ± 0.4	3.3 ± 0.3
Cerebral perfusion pressure, mmHg	75 ± 6	66 ± 4	68 ± 8	63 ± 3
Pressure reactivity index	−0.3 ± 0.1	−0.2 ± 0.1	−0.1 ± 0.1	−0.2 ± 0.1
SjvO_2_, %	76 ± 7	79 ± 5	76 ± 6	76 ± 7
Heart rate, bpm	105 ± 14	113 ± 15	89 ± 5	121 ± 18
Mean arterial pressure, mmHg	93 ± 6	79 ± 5	84 ± 7	82 ± 4
Cardiac index, ml/kg/min	87 ± 7	84 ± 5	98 ± 7	98 ± 6
Lactate, mmol/l	3.4 ± 0.1	3.4 ± 0.3	2.8 ± 0.4	3.2 ± 0.4
Arterial blood pH	7.45 ± 0.01	7.43 ± 0.02	7.47 ± 0.02	7.44 ± 0.01
PaO_2_, mmHg	156 ± 8	156 ± 9	162 ± 3	154 ± 9
PaCO_2_, mmHg	41 ± 1	40 ± 2	40 ± 1	42 ± 1
Bicarbonate, mmol/l	27 ± 1	26 ± 1	28 ± 1	27 ± 1
Hematocrit, %	26 ± 1	27 ± 1	25 ± 1	27 ± 1
**CPR characteristics**				
ROSC time (resuscitated animals), min	14 ± 1	15 ± 1	32 ± 0	35 ± 1
Coronary perfusion pressure, mmHg	6 ± 2	7 ± 2	2 ± 1	4 ± 1
Electric shocks (all animals), *n*	4 ± 2	2 ± 1	3 ± 1	6 ± 2
Electric shocks (resuscitated animals), *n*	4 ± 2	2 ± 1	1 ± 0	4 ± 2
Epinephrine bolus (all animals), *n*	1 ± 0	−	2 ± 1	−
Epinephrine bolus (resuscitated animals), *n*	1 ± 0	−	1 ± 0	−

Data are expressed as mean ± SEM. Student *t*-test found no statistically significant differences between the CCPR 10′ and ECPR 10′ groups, or between the CCPR 30′ and ECPR 30′ groups.

*CCPR, conventional cardiopulmonary resuscitation; CPR, cardiopulmonary resuscitation; ECPR, extracorporeal cardiopulmonary resuscitation; PaCO_2_, arterial carbon dioxide partial pressure; PaO_2_, arterial oxygen partial pressure; ROSC, return of spontaneous circulation; SjvO_2_, jugular venous oxygen saturation*.

### Cerebral hemodynamics and oxygenation

**Fig. 2:**
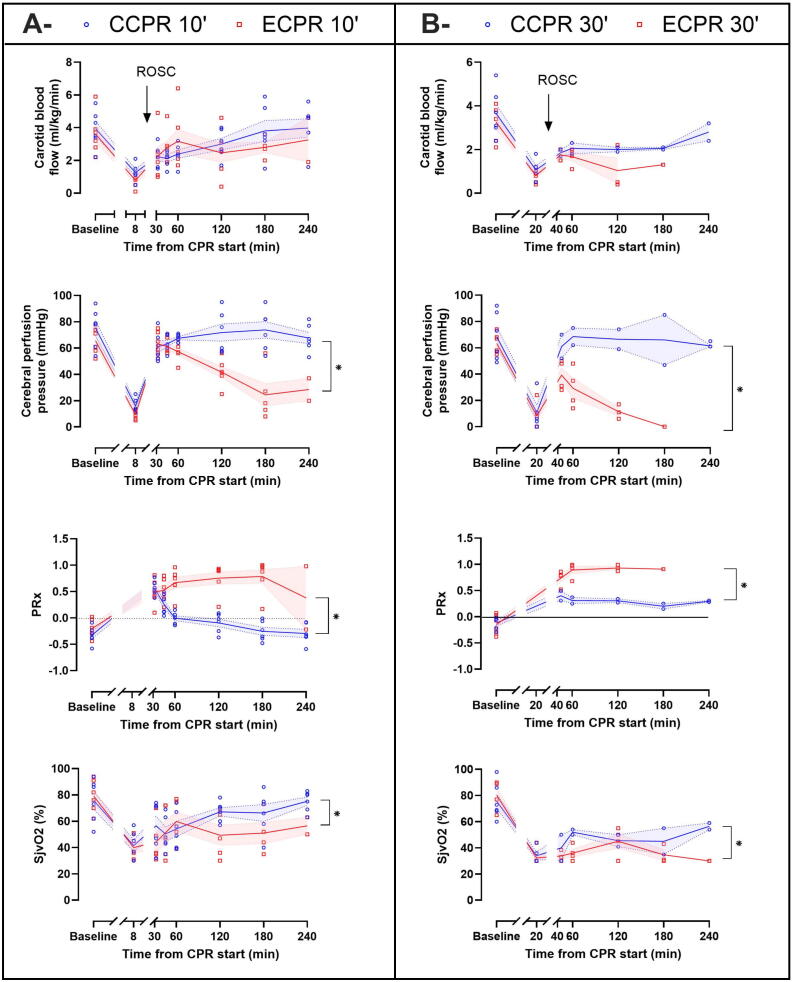
**Cerebral hemodynamics and oxygenation parameters comparison between CCPR 10′ and ECPR 10′ groups (A) and CCPR 30′ and ECPR 30′ groups (B) after cardiopulmonary resuscitation (CPR). In CCPR 10′ and ECPR 10′ groups, values are presented at *t* = 8 min (initial CPR phase, before defibrillation attempts) or after achievement of resumption of spontaneous circulation (ROSC) at = 35, 45, 60, 120, 180, 240 min in surviving animals. In CCPR 30′ and ECPR 30′ groups, values are presented at *t* = 20 min (initial CPR phase) or after ROSC at *t* = 45, 60, 120, 180 and 240 min in surviving animals**. Symbols represent sample size and dispersion at each time of interest (CCPR and ECPR in blue and red, respectively). Lines and error bands represent means and SEM respectively. *P < 0.05 for the group effect of the mixed model for repeated measures for post-ROSC analysis, whose *P-values* of the contingency tables are presented in Table S1 (CCPR 10′ vs ECPR 10′) and Table S2 (CCPR 30′ vs ECPR 30′). *CCPR, conventional cardiopulmonary resuscitation; CPR, cardiopulmonary resuscitation; ECPR, extracorporeal cardiopulmonary resuscitation; PRx, pressure reactivity index; SjvO_2_, jugular venous oxygen saturation*. (For interpretation of the references to color in this figure legend, the reader is referred to the web version of this article.)

Fig. 2 illustrates key cerebral hemodynamics and oxygenation parameters. No significant difference was observed at baseline or after CPR initiation before ROSC (i.e., *t* = 8 min in CCPR 10′ and ECPR 10′ groups and *t* = 20 min in CCPR 10′ and ECPR 10′ groups, respectively). After ROSC, CBF was neither significantly different between the CCPR 10′ and ECPR 10′ groups, nor between the CCPR 30′ and ECPR 30′ groups. Although there was no statistically significant difference in ICP, CePP was significantly higher in the CCPR 10′ and the CCPR 30′ groups as compared with the ECPR 10′ and ECPR 30′ groups, respectively. Cerebral vascular resistances were higher in the CCPR 10′ group as compared to the ECPR 10′ group but not in the CCPR 30′ group as compared with the ECPR 30′ group. Regarding cerebral oxygenation, a significant increase in SjvO_2_ was observed in the CCPR 10′ group as compared to the ECPR 10′ group and in the CCPR 30′ group as compared to the ECPR 30′ group. Finally, altered cerebral autoregulation, reflected by significantly greater PRx was found in ECPR 10′ and ECPR 30′ groups, in comparison to CCPR 10′ and CCPR 30′ groups, respectively (Tables S1 and S2).

### Systemic hemodynamics

**Fig. 3:**
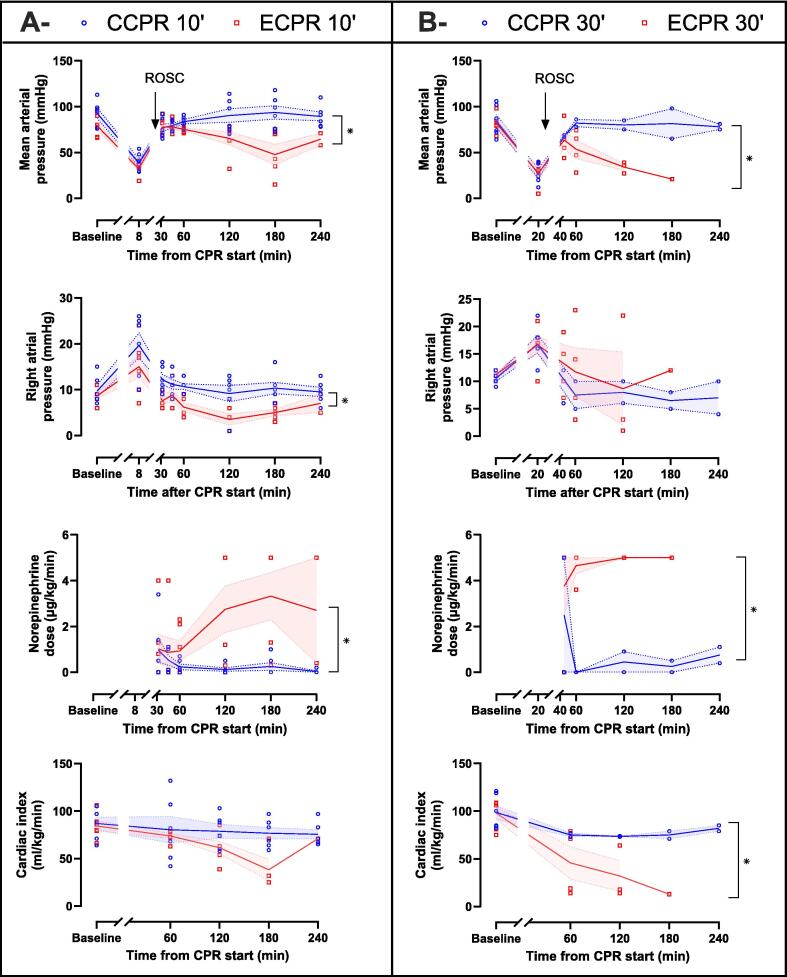
Systemic hemodynamics parameters comparison between CCPR 10′ and ECPR 10′ groups (A) and CCPR 30′ and ECPR 30′ groups (B) after cardiopulmonary resuscitation (CPR). In CCPR 10′ and ECPR 10′ groups, values are presented at = 8 min (initial CPR phase, before defibrillation attempts) or after achievement of resumption of spontaneous circulation (ROSC) at = 35, 45, 60, 120, 180, 240 min in surviving animals. In CCPR 30′ and ECPR 30′ groups, values are presented at *t* = 20 min (initial CPR phase) or after ROSC at *t* = 45, 60, 120, 180 and 240 min in surviving animals. Symbols represent sample size and dispersion at each time of interest (CCPR and ECPR in blue and red, respectively). Lines and error bands represent means and SEM respectively. *P < 0.05 for the group effect and ^†^*P* < 0.05 for the time × group interaction of the mixed model for repeated measures for post-ROSC analysis, whose *P*-values of the contingency tables are presented in Table S1 (CCPR 10′ vs ECPR 10′) and Table S2 (CCPR 30′ vs ECPR 30′). *CCPR, conventional cardiopulmonary resuscitation; CPR, cardiopulmonary resuscitation; ECPR, extracorporeal cardiopulmonary resuscitation*. (For interpretation of the references to color in this figure legend, the reader is referred to the web version of this article.)

Fig. 3 shows the main systemic hemodynamic parameters. No significant difference was observed at baseline or before ROSC (i.e., *t* = 8 min in CCPR 10′ and ECPR 10′ groups and *t* = 20 min in CCPR 10′ and ECPR 10′ groups, respectively). After ROSC, CCPR 10′ and CCPR 30′ groups were associated with a significant increase in MAP despite a much lower norepinephrine dose, as compared with ECPR 10′ and ECPR 30′ groups, respectively. Right atrial pressure was higher in CCPR 10′ vs ECPR 10′ and not different between CCPR 30′ vs ECPR 30′. The cardiac index was significantly greater in the CCPR 30′ group vs ECPR 30′ but not in CCPR 10′ vs ECPR 10′. Finally, CCPR 10′ group had a significantly lower heart rate as well as a higher RAP, pulse pressure and central venous oxygen saturation than the ECPR 10′ group, but these differences were not found between CCPR 30′ and ECPR 30′ groups (Tables S1 and S2).

### Blood biochemistry

**Fig. 4:**
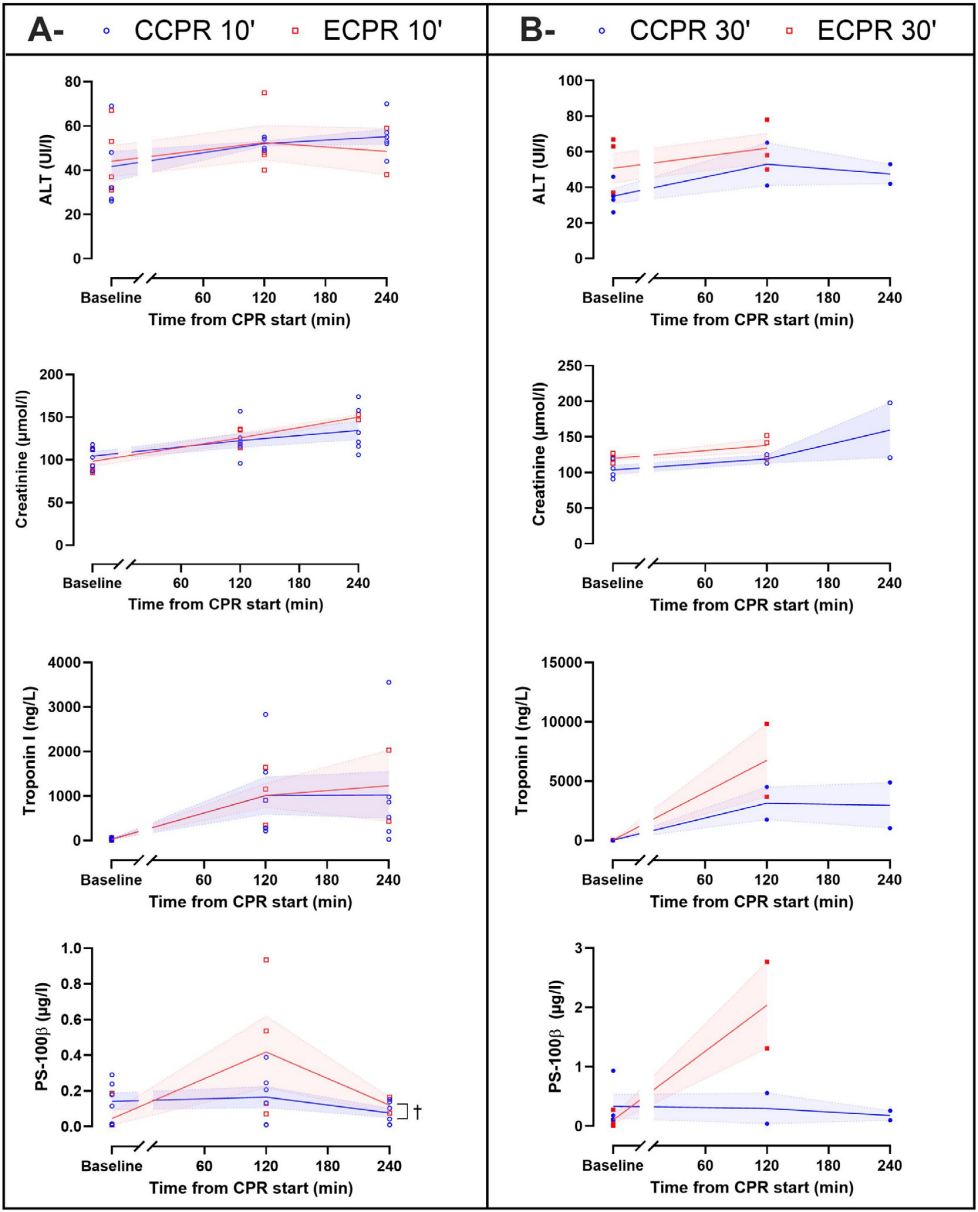
**Multiorgan failure biochemical markers comparison between CCPR 10′ and ECPR 10′ groups (A) and CCPR 30′ and ECPR 30′ groups (B) after cardiopulmonary resuscitation (CPR *t* = 120 and 240 min) start in surviving animals**. Symbols represent sample size and dispersion at each time of interest. Lines and error bands represent means and SEM respectively. ^†^*P* < 0.05 for the time × group interaction of the mixed model for repeated measures for post-ROSC analysis, whose *P*-values of the contingency tables are presented in Table S1 (CCPR 10′ vs ECPR 10′) and Table S2 (CCPR 30′ vs ECPR 30′). *ALT, alanine aminotransferase; CCPR, conventional cardiopulmonary resuscitation; CPR, cardiopulmonary resuscitation; ECPR, extracorporeal cardiopulmonary resuscitation; PS-100β, protein S-100β.*

Fig. 4 illustrates multiorgan failure biochemical markers. Blood levels of PS-100β, indicating neuronal damages, were significantly higher in the ECPR 10′ group as compared with the CCPR 10′ group but not in the ECPR 30′ group as compared with the CCPR 30′ group. In contrast, blood tests of ALT, creatinine and troponin I was comparable between groups (Tables S1 and S2).

**Fig. 5:**
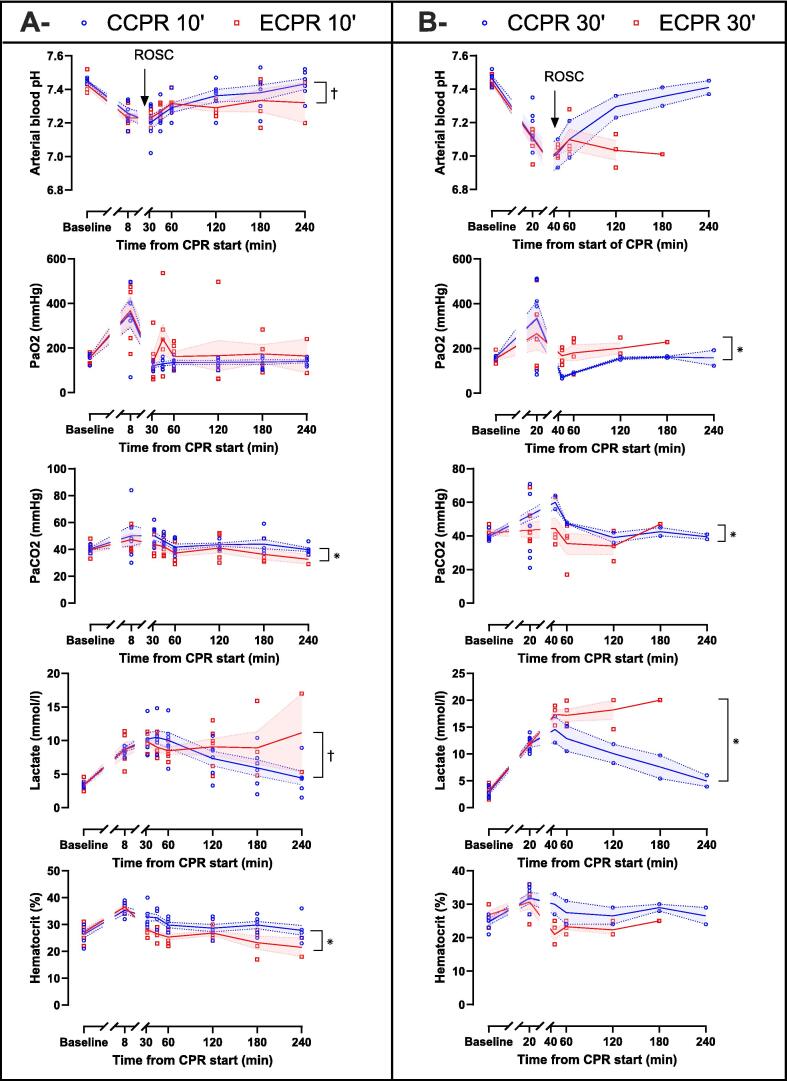
**Arterial blood gases, lactate blood levels and hematocrit in CCPR 10′ and ECPR 10′ groups (A) and CCPR 30′ and ECPR 30′ groups (B) after cardiopulmonary resuscitation (CPR) start. In CCPR 10′ and ECPR 10′ groups, values are presented at = 8 min (initial CPR phase, before defibrillation attempts) or after achievement of resumption of spontaneous circulation (ROSC) at = 35, 45, 60, 120, 180, 240 min in surviving animals. In CCPR 30′ and ECPR 30′ groups, values are presented at *t* = 20 min (initial CPR phase) or after ROSC at *t* = 45, 60, 120, 180 and 240 min in surviving animals**. Symbols represent sample size and dispersion at each time of interest. Lines and error bands represent means and SEM respectively. *P < 0.05 for the group effect and ^†^*P* < 0.05 for the time × group interaction of the mixed model for repeated measures for post-ROSC analysis, whose *P*-values of the contingency tables are presented in Table S1 (CCPR 10′ vs ECPR 10′) and Table S2 (CCPR 30′ vs ECPR 30′). *CCPR, conventional cardiopulmonary resuscitation; CPR, cardiopulmonary resuscitation; ECPR, extracorporeal cardiopulmonary resuscitation; PaCO_2_ arterial carbon dioxide partial pressure; PaO_2_, arterial oxygen partial pressure*.

Others biological parameters are shown in Fig. 5. No significant difference was observed at baseline or before ROSC (i.e., *t* = 8 min in CCPR 10′ and ECPR 10′ groups and *t* = 20 min in CCPR 10′ and ECPR 10′ groups, respectively). After ROSC, CCPR 10′ group had less pronounced metabolic acidosis, with higher arterial blood pH and bicarbonate levels values than the ECPR 10′ group. PaCO_2_ was significantly higher in the CCPR 10′ and CCPR 30′ groups as compared to the ECPR 10′ and ECPR 30′ groups, respectively. PaO_2_ was significantly lower in the CCPR 30′ group vs ECPR 30′ group but not in CCPR 10′ vs ECPR 10′. Blood lactate level was significantly lower in the CCPR 10′ and CCPR 30′ groups, as compared to the ECPR 10′ and ECPR 30′ groups, respectively. Finally, hematocrit was greater in the CCPR 10′ group than the ECPR 10′ group, with no difference found between CCPR 30′ and ECPR 30′ (Tables S1 and S2).

## Discussion

In a swine model of CA, an early ECPR strategy was associated with impaired systemic and cerebral hemodynamics and oxygenation after ROSC when started systematically vs CCPR with epinephrine. The pattern of alterations was similar when ECPR was started after either a 10- or 30-min low-flow vs CCPR. It was evidenced by lower CePP and SjvO_2_, as well as higher PRx in ECPR 10′ and ECPR 30′ groups after ROSC, as compared to CCPR 10′ and CCPR 30′ groups, respectively. ECPR was also related to lower MAP, as well as higher norepinephrine dose and blood lactate levels.

Importantly, the design of the present study model was chosen in line with clinical practice, since in humans, it is recommended to consider the deployment of ECPR and start cannulation in case of short no-flow time (≤5 min) and failure of an optimal CCPR of sufficient duration.^5^, ^6^ However, the main assumption that could explain the global alteration of systemic and cerebral hemodynamics and oxygenation with ECPR is that brain and myocardial injury was not severe enough. Indeed, ECPR was initiated ultra-early and systematically before CCPR was unsuccessful in achieving ROSC. Consequently, the systematic implementation of peripheral veno-arterial ECMO in these pigs without major cardiac impairment may have been futile and even deleterious given its non-physiological hemodynamic impact. The side effect of ECMO could then involve increased left ventricular afterload due to competitive retrograde ECMO blood flow, reduced right ventricular preload because of impaired venous return or non-pulsatile flow.^17^ Longer time to ROSC or increased number of defibrillation attempts could also worsen cardiac injury in ECPR 30′ vs CCP30′. Our results should therefore be interpreted in the light of the CA model design, including a short no-flow (5 min) and an intermediate low-flow duration (10 or 30 min, according to experimental groups). Interestingly, in most previous experimental studies, ECPR was started after prolonged VF (median duration: 15 min)^18^ and little period or lack of low-flow (i.e., chest compressions and mechanical ventilation), which is less consistent with clinical practice.^14^ As an example, Yan et al. aimed to compare CCPR and ECPR in a swine model of CA with a 12-min no-flow and a 2-min low-flow.^19^ In this study, the authors reported a benefit of ECPR on survival, time to ROCS achievement and systemic hemodynamics, evidenced by lower heart rate and higher MAP and cardiac output.^19^

This discrepancy between these data and our findings could be explained by the deployment of ECPR at different phases of the CA and resuscitation process. According to the 3-phase time-sensitive model of CA from VF, three successive stages can indeed be described: (i) the electrical phase (until 4 min from VF start), requiring prompt defibrillation; (ii) the circulatory phase (4–10 min from VF start), with urgent need to restore blood flow with chest compressions first; and iii) the metabolic phase, where both chest compressions and defibrillation are no longer sufficient to achieve sustained ROSC.^20^ From this theoretical perspective, ECPR might be only beneficial in “metabolic” CA models with prolonged no-flow, supporting the complexity of its assessment, which must be neither too early (when ROSC occurrence is the goal and can still be achieved with chest compressions and defibrillation) nor too late, to expect benefits in post-resuscitation neurological status. This hypothesis is supported by a recent experimental study by Moreau et al. in which the authors found no difference in norepinephrine dose or blood lactate level following ROSC between pigs submitted to ECPR after either 18-min no-flow/12-min low-flow (model 1, *n* = 5) or 5-min no-flow/25-min low-flow (model 2, *n* = 5). This lack of improvement in systemic hemodynamics in the second “circulatory” model of CA despite a much shorter no-flow time is an argument in line with the assumption that benefit of ECPR mainly concerns CA in the metabolic phase with prolonged no-flow after which pigs would not achieve ROSC with CCPR alone.^21^ The benefit is then only related to the resuscitation capabilities but not the hemodynamic support after ROSC. However, this statement is not directly supported by our results since ECPR did not increase ROSC rate in our study. Conversely, one might also argue that ECPR modalities should be improved to facilitate ROSC and prevent its putative deleterious effect after ROSC using e.g., non-continuous flow, higher flow targets or dedicated therapies preventing capillary leaks.

Alternative hypotheses may also be raised to explain the hemodynamic impairment with ECPR after ROSC in our study. Firstly, epinephrine administration, performed only in the CCPR 10′ and 30′ groups, could have a beneficial effect regarding the relatively low doses that were administered, as reported in most contemporary experimental investigations in swine models of CA^22^, ^23^ and one prospective observational study in 36 patients.^24^ Then, ECPR could directly affect cerebral autoregulation, due to the non-pulsatile flow of veno-arterial ECMO and its impairment on nitric oxide basal production, as demonstrated in healthy newborns lambs.^25^, ^26^ Moreover, the much higher doses of norepinephrine required after ROSC in the ECPR 10′ and 30′ groups might have a deleterious effect to the cerebral microvascular bed through increased resistance to cortical blood supply.^27^ Finally, ECPR may increase post-resuscitation brain injury, as oxygenated blood flow is quickly restored after sustained hypoxia.^2^

### Study limitations

Our study presents several limitations. Firstly, the small sample number of animals included may have reduced statistical power and limit the generalizability of our findings, especially for the comparison between CCPR 30′ and ECPR 30′ groups. Secondly, the short duration of animal follow-up (4 h) prevented a multimodal neurological assessment of major prognostic interest (clinical, electrophysiological, imaging, histological, etc.).^2^ Consequently, we can only draw conclusions on surrogate markers such as PS-100β, CePP (global cerebral hemodynamics parameter), PRx (cerebral autoregulation indicator)^16^ or SjvO_2_ (reflecting the balance between cerebral oxygen delivery and consumption).^28^ Nevertheless, impairment in cerebral hemodynamics and oxygenation is common after CA in humans,^29^ and alterations in CePP,^30^ PRx^31^, ^32^, ^33^ or SjvO_2_,^34^ are associated with poor neurological outcomes in clinical research. Thirdly, we set the target flow rate at 35 ml/kg based on preliminary data showing that higher flows cannot be achieved without much higher fluid volumes than allowed here. One would argue that the protocol should have allowed higher amounts of fluid to target greater ECMO flows but that this was associated with major capillary leaks with abdominal fluid collection in preliminary experiments. However, we cannot rule out that higher ECMO flow target, even if requiring greater fluid amount, could have led to improved cerebral and systemic hemodynamics. Fourthly, it is important to note that in ECPR 10′ and ECPR 30′ groups, ECMO circuits were primed with Ringer’s lactate solution. In the absence of a volume correction strategy (e.g., transfusion, diuretics, hemofiltration), this inevitably led to hemodilution in ECPR 10′ and ECPR 30′ groups and may have contributed to the impairment in cerebral oxygenation observed in these groups, when compared to the CCPR 10′ and CCPR 30′ groups. Therefore, our results cannot be generalized to blood-primed ECPR or subsequent transfusion during ECMO support. Fifthly, some significant differences between groups in parameters of known neurological impact after CA, such as PaCO_2_, may have constituted a confounding factor in the comparison of cerebral hemodynamics and SvjO_2_.^2^ Transient hyperoxemia in some animals with ECPR could also worsen the brain injury. Finally, our experimental model presents several notable differences from humans. Indeed, the lack of amiodarone administration in CCPR 10′ and 30′ groups, despite its recommendation in CA with shockable rhythm after three shocks delivery,^5^, ^6^ or the immediate application of an “ideal” CPR, rarely performed by bystanders in out-of-hospital setting, differ from real-life clinical practice. Additionally, chest compression without medications or defibrillation until 10 min after initiation of CPR does not emulate in-hospital arrest where time to defibrillation is likely to be shorter. Conversely, implementing ECPR very early after out-of-hospital cardiac arrest poses the greatest challenge. The fact that only females were included is another notable limitation, since they have lower mortality, a better neurological prognosis and a reduced systemic inflammatory response as compared with males.^35^ Likewise, certain specific features of experimental studies, such as the control of conditions preceding the induction of CA (e.g., temperature, anesthesia, fasting, etc.) could play a neuroprotective role^35^ and limit the extrapolation of study results.

## Conclusions

In a swine model of CA, early and systematic crystalloid-primed ECPR implemented after either a 10- or 30-min low-flow period resulted in impaired systemic and cerebral hemodynamics after ROSC, compared with to CCPR with epinephrine. However, our conclusions could not necessarily be extrapolated to other conditions of ECPR, such as blood-primed circuits, higher ECMO flow targets or more liberal fluid management. Further studies are needed with systematic ECPR after cardiac arrest in these settings.

## Funding

The study was funded by Agence Nationale pour la Recherche (Grant Areg-shock ANR-21-CE17-0011-03).

## CRediT authorship contribution statement

**Julian San Geroteo:** Writing – review & editing, Writing – original draft, Methodology, Investigation, Conceptualization. **Ali Jendoubi:** Writing – review & editing, Methodology, Investigation. **Fanny Lidouren:** Writing – review & editing, Methodology, Investigation. **Naoto Watanabe:** Writing – review & editing, Methodology, Investigation. **Yara Abi Zeid Daou:** Writing – review & editing, Methodology, Investigation. **Alice Hutin:** Writing – review & editing, Methodology, Conceptualization. **Lionel Lamhaut:** Writing – review & editing, Methodology, Conceptualization. **Nadir Mouri:** Writing – review & editing, Investigation. **Bijan Ghaleh:** Writing – review & editing. **Pierre-Louis Léger:** Writing – review & editing, Methodology, Conceptualization. **Jerome Rambaud:** Writing – review & editing, Methodology, Conceptualization. **Rebecca Goutchtat:** Writing – review & editing, Methodology, Investigation, Conceptualization. **Matthias Kohlhauer:** Writing – review & editing, Methodology, Investigation, Conceptualization. **Renaud Tissier:** Writing – review & editing, Writing – original draft, Validation, Supervision, Project administration, Investigation, Funding acquisition, Formal analysis, Data curation, Conceptualization.

## Ethics approval

All experiments were reviewed and approved by the ethical committee ComEth Anses‑EnvA‑UPEC (Committee No. 16, project APAFIS #47825-2024022914398265 v4).

## Funding

The authors have stated they have no financial relationships relevant to this article to disclose.

## Declaration of competing interest

RT and MK are shareholders of a start-up company dedicated to total liquid ventilation (Orixha).
