# Supplementary material for: Evaluation of systemic and cerebral hemodynamics after systematic and early extracorporeal cardiopulmonary resuscitation in swine
Source: Resusc Plus. 2026 Jan 18;28:101233. doi: 10.1016/j.resplu.2026.101233 (PMC12874813; doi:10.1016/j.resplu.2026.101233)
Supplement: Supplementary Tables S1 and S2 [file mmc1.pdf]

SUPPLEMENTARY INFORMATION

Supplementary Material 1.

Table S1: P-values of the contingency table for the comparison of investigated parameters after resuscitation between CCPR 10’ and ECPR 10’ groups using mixed model for repeated measures with time, group and time x group interaction effects.

	**P value**			***In case of sign. group effect or interaction***	
	**Time Effect**	**Group Effect**	**Time x Group**	**Diff. between predicted means (CCPR10’ – ECPR10’)**	**SE of difference**
**Clinical characteristics**					
Bladder temperature, °C	0.72	<0.01*	0.30	1.92	0.33
**Cerebral hemodynamics**					
Carotid blood flow, ml/kg/min	0.07	0.55	0.11	-	-
Intracranial pressure, mmHg	0.01*	0.25	0.06	-	-
Cerebral perfusion pressure, mmHg	0.06	<0.01*	<0.01*	25.65	3.76
Pressure reactivity index	<0.01*	<0.01*	<0.01*	-0.65	0.11
Cerebral vascular resistance, woods unit	0.01*	0.73	0.04*	0.08	0.23
**Cerebral oxygenation**					
Jugular venous oxygen saturation, %	0.29	0.03*	0.30	11.86	4.84
**Systemic hemodynamics**					
Heart rate, bpm	0.24	0.01*	0.83	-35.69	11.90
Right atrial pressure, mmHg	0.04*	<0.01*	0.61	4.54	1.18
Mean arterial pressure, mmHg	0.47	<0.01*	<0.01*	22.00	5.50
Norepinephrine dose, µg/kg/min	0.04*	0.02*	<0.01*	-2.00	0.70
Pulse pressure, mmHg	0.02*	0.94	<0.01*	0.26	3.51
Cardiac index, ml/kg/min	0.04*	0.07	0.12	-	-
Lactate, mmol/l	0.07	0.19	<0.01*	-2.08	1.47
**Blood biochemistry**					
Arterial blood pH	<0.01*	0.46	0.03*	0.03	0.04
PaO_2, mmHg	0.72	0.39	0.99	-	-
$PaCO_2$, mmHg	0.02*	0.04*	0.81	5.95	2.48
Bicarbonate, mmol/l	<0.01*	0.04*	<0.01*	4.20	1.82
Hematocrit, %	<0.01*	0.03*	0.15	4.28	1.66
Alanine aminotransferase, UI/l	0.16	0.81	0.74	-	-
Creatinine, µmol/l	<0.01*	0.65	0.45	-	-
Troponin I, ng/l	0.03*	0.84	0.96	-	-
Protein S-100β, µg/l	0.01*	0.42	0.04*	-0.08	0.09

Parameters were measured after CPR start. *P <0.05 was considered statistically significant.

CCPR, conventional cardiopulmonary resuscitation; CDO_2, cerebral oxygen delivery; $CMRO_2$, cerebral metabolic rate of oxygen; CO_2 gap, central venous-to-arterial CO_2 difference; CPR, cardiopulmonary resuscitation; ECPR, extracorporeal cardiopulmonary resuscitation; $PaCO_2$ arterial carbon dioxide partial pressure; PaO_2, arterial oxygen partial pressure.

Supplementary Material 2.

Table S2: P-values of the contingency table for the comparison of investigated parameters after resuscitation between CCPR 30’ and ECPR 30’ groups using mixed model for repeated measures with time and group effects.

	P value		***In case of sign. group effect***	
	**Time Effect**	**Group Effect**	**Diff. between predicted means (CCP10’ – ECPR10’)**	**SE of difference**
**Clinical characteristics**				
Bladder temperature, °C	0.16	0.02*	1.26	0.32
**Cerebral hemodynamics**				
Carotid blood flow, ml/kg/min	0.27	0.42	-	-
Intracranial pressure, mmHg	0.63	0.26	-	-
Cerebral perfusion pressure, mmHg	0.27	0.02*	50.62	12.43
Pressure reactivity index	0.61	<0.01*	-0.63	0.05
Cerebral vascular resistance, woods unit	0.26	0.06	-	-
**Cerebral oxygenation**				
Jugular venous oxygen saturation, %	0.18	0.03*	10.64	3.63
**Systemic hemodynamics**				
Heart rate, bpm	0.17	0.06	-	-
Right atrial pressure, mmHg	0.38	0.44	-	-
Mean arterial pressure, mmHg	0.30	0.02*	43.13	11.81
Norepinephrine dose, µg/kg/min	0.47	<0.01*	-3.00	0.77
Pulse pressure, mmHg	0.26	0.74	-	-
Cardiac index, ml/kg/min	0.51	0.04*	44.00	15.00
Lactate, mmol/l	0.12	0.02*	-6.35	1.71
**Blood biochemistry**				
Arterial blood pH	0.13	0.14	-	-
PaO_2, mmHg	0.23	<0.01*	-72.44	20.41
$PaCO_2$, mmHg	0.06	0.02*	10.68	4.15
Bicarbonate, mmol/l	0.18	0.06	-	-
Hematocrit, %	0.50	0.12	-	-
Alanine aminotransferase, UI/l	0.23	0.12	-	-
Creatinine, µmol/l	0.04*	0.19	-	-
Troponin I, ng/l	0.05	0.36	-	-
Protein S-100β, µg/l	0.11	0.29	-	-

The time x group interaction was not evaluated in this model, due to the low number of resuscitated animals. Parameters were measured after CPR start. **P* <0.05 was considered statistically significant.

CCPR, conventional cardiopulmonary resuscitation; CDO_2, cerebral oxygen delivery; $CMRO_2$, cerebral metabolic rate of oxygen; CO_2 gap, central venous-to-arterial CO_2 difference; CPR, cardiopulmonary resuscitation; ECPR, extracorporeal cardiopulmonary resuscitation; $PaCO_2$ arterial carbon dioxide partial pressure; PaO_2, arterial oxygen partial pressure.
